# Integrated transcriptomic and metabolomic analysis reveals the regulatory role of itaconic acid in inflammatory infiltration during myocardial ischemia-reperfusion injury

**DOI:** 10.7717/peerj.21525

**Published:** 2026-07-17

**Authors:** QuanWei Pei, ZeXin Zhou, SiJia Dong, QiQi Shao, Fen Liu, ZhenYan Fu, YiTong Ma

**Affiliations:** 1 Heart Center, First Affiliated Hospital of Xinjiang Medical University, Xinjiang Medical University, Urumqi, XinJiang, China; 2 Clinical Medical Research Institute, The First Affiliated Hospital of Xinjiang Medical University, Xinjiang Medical University, Urumqi, China

**Keywords:** Myocardial ischemia-reperfusion, Itaconic acid, Transcriptomics, Metabolomics, Inflammation

## Abstract

**Background:**

Inflammatory infiltration constitutes a fundamental pathophysiological mechanism in myocardial ischemia-reperfusion (IR) injury, characterized by its role in initiating tissue damage, amplifying pathological inflammatory cascades, and exacerbating structural and functional impairment of the myocardium. Integrated transcriptomic and metabolomic analysis identified the metabolite itaconic acid as a potential key regulator in IR injury. Therefore, we assessed the effect of its derivative, 4-octyl itaconate (4-OI), on inflammatory infiltration in a mouse model of IR injury.

**Methods:**

Following establishment of a myocardial IR model, tissue samples from the infarct border zone were harvested for transcriptomics and wide-target metabolomic sequencing. Bioinformatics methods were used to analyze the transcriptomics and metabolomics results separately and to perform a joint analysis. Molecular docking and molecular dynamics modeling were employed to explore proteins bound to itaconic acid. Following intragastric administration of 4-OI to myocardial IR mice, echocardiography was performed. Plasma levels of interleukin-4 (IL4), interleukin10 (IL10), cardiac troponin T (cTnT), and creatine kinase-myocardial band (CKMB) were measured by enzyme-linked immunosorbent assay (ELISA). Myocardial inflammatory infiltration was evaluated by hematoxylin and eosin Staining (HE staining), while inflammatory marker expression associated with macrophages was evaluated by immunohistochemical staining for inducible nitric oxide synthase (iNOS) and immune responsive gene 1 (IRG1). Macrophage heterogeneity was further assessed by immunofluorescence co-localization staining for F4/80, CCR2, and CD206. Additionally, mRNA expression levels of Interleukin-1 beta (Il1b), Tumor Necrosis Factor-alpha (Tnfa), Il4, and Il10 in myocardial tissue were quantified by quantitative reverse transcription polymerase chain reaction (qRT-PCR).

**Results:**

Integrated transcriptomic and metabolomic analysis identified itaconic acid as a potential metabolite modulating myocardial IR injury. Preliminary molecular docking analysis suggested possible *in silico* interactions between itaconic acid and Pla2g2d, Lcn2, and Gpr55 proteins, which require further experimental validation. A derivative of itaconic acid, 4-OI, significantly ameliorated myocardial IR injury in mice, reduced inflammatory infiltration, decreased iNOS-associated staining, and modestly increased Arg1-associated staining in the infarct border zone. This treatment was also accompanied by differences in macrophage marker-defined populations, reflected by fewer F4/80^+^CCR2^+^ and more F4/80^+^CD206^+^ cells in the injured myocardium.

## Introduction

Ischemic heart disease is one of the most prevalent and deadly diseases worldwide, posing a significant threat to public health (Tsao et al., 2022). Prompt reperfusion therapy (such as percutaneous coronary intervention) for patients with acute myocardial infarction is an effective means of restoring blood supply to ischemic myocardium and has become the standard clinical treatment for myocardial infarction. Although recent advancements in revascularization techniques have significantly reduced short-term mortality in myocardial infarction patients, reperfusion itself can induce additional myocardial cell death and irreversible myocardial damage, a phenomenon known as ischemia-reperfusion (IR) injury (Xiang et al., 2024). Following myocardial IR injury, excessive inflammatory infiltration represents a key pathological event contributing to secondary myocardial damage. Its detrimental effects extend beyond immediate infarct expansion, driving a persistent imbalance between inflammation and repair that ultimately leads to adverse cardiac remodeling and heart failure (Wang et al., 2025).

Itaconic acid is a metabolite derived from the tricarboxylic acid (TCA) cycle intermediate cis-aconitate, generated by decarboxylation catalyzed by aconitate decarboxylase 1 (ACOD1), which is encoded by immune responsive gene 1 (IRG1). Upon entering cells *via* the transporter SLC13A3, itaconic acid induces transcription factor EB (TFEB)-dependent lysosomal biogenesis, thereby enhancing antibacterial innate immunity in hepatocytes (Liu et al., 2025b). Mills et al. (2018) first reported that 4-OI, a derivative of itaconic acid, limits inflammatory responses in macrophages during sepsis through the Nrf2 pathway and modulates the type I interferon negative feedback loop. In addition, itaconic acid regulates immune responses in various cell types, including T cells, neutrophils, hepatocytes, and neural cells (McGettrick et al., 2024). In the field of myocardial IR injury, protective effects of 4-OI have been demonstrated. Yang et al. (2025) showed that 4-OI promotes vascular endothelial cell proliferation and migration *via* activation of the MAPK/ERK signaling pathway, enhances angiogenesis in ischemic regions, and improves post-injury cardiac repair. Another study revealed that 4-OI alleviates myocardial IR injury after heart transplantation by inhibiting neutrophil extracellular trap (NET) formation, and reduces cardiomyocyte apoptosis by covalently modifying Cys474, Cys424, and Lys151 of pyruvate kinase M2 (PKM2), facilitating PKM2 translocation to the mitochondria and reducing Bcl2 degradation (Tian et al., 2025). Furthermore, a drug delivery system combining 4-OI as an Nrf2 activator with a mitochondria-protective strategy reduces reactive oxygen species (ROS) burst, improves mitochondrial function, and attenuates myocardial IR injury (Liao et al., 2025). In contrast to the aforementioned anti-inflammatory and protective effects, some studies have shown that itaconic acid promotes the release of pro-inflammatory cytokines (IL6, IL1b) and enhances NLRP3 inflammasome activation in alveolar macrophages (Shan et al., 2025a), suggesting that the immunomodulatory function of itaconic acid under specific pathological conditions requires further investigation. In myocardial IR injury, inflammatory infiltration, macrophage heterogeneity, angiogenic repair, and ERK signaling are not isolated processes but are biologically interconnected components of the post-ischemic repair response. Given that macrophages, particularly those with a reparative phenotype (*e.g.*, F4/80^+^CD206^+^ macrophages), are known to secrete pro-angiogenic factors such as VEGF, it is plausible that immunometabolic reprogramming of macrophages by 4-OI may act upstream of or in parallel with the ERK-mediated angiogenic effects reported by Yang et al. However, whether 4-OI modulates inflammatory infiltration through regulation of macrophage heterogeneity remains unclear. Therefore, building upon the established protective effects of 4-OI, the present study explores whether 4-OI administration is associated with changes in inflammatory infiltration and macrophage heterogeneity following myocardial IR injury, with the goal of generating hypotheses regarding its possible cardioprotective mechanisms.

## Materials & Methods

### Animals

All male C57BL/6J mice were purchased from the Animal Experiment Center of Xinjiang Medical University, the mice were subjected to experimentation after 7 days of acclimatization, and housed in individually ventilated cage systems under controlled conditions with a room temperature of 22–24 °C, relative humidity of 40–60%, and a 12/12-hour light–dark cycle. They had free access to standard breeding feed and autoclaved drinking water, with no more than five mice per cage to ensure sufficient living space. For environmental enrichment, each cage was supplied with sterile bedding. During the myocardial IR model establishment, anesthesia was induced using isoflurane. Deep anesthesia and euthanasia were achieved by intraperitoneal injection of pentobarbital sodium (150 mg/kg). Tissue collection proceeded after confirming cardiac arrest and pupillary dilation. All procedures complied with the NIH Guide for the Care and Use of Laboratory Animals and were approved by the Institutional Ethics Committee of the First Affiliated Hospital of Xinjiang Medical University under approval number 240301-154. For the mouse model of myocardial IR injury, pre-established humane endpoints requiring euthanasia before the planned end of the experiment included: (1) severe respiratory distress; (2) inability to access food or water; (3) signs of prolonged (>12 h) severe pain or distress (*e.g.*, continuous vocalization, self-mutilation); (4) >20% body weight loss within 24 h; or (5) development of paralysis or severe neurological deficits.

The experimental design employed a randomized grouping method (using a computer-generated random sequence to assign mice stratified by body weight to the sham operation group, myocardial IR model group, and myocardial IR with 4-OI supplementation group), with blinded assessment implemented (the personnel assessing the outcomes were unaware of the group assignments). Mice grouping and numbers were determined based on existing literature.

### Cardiac ischemia reperfusion injury

Mice (male, 7–8 weeks old, 23–25 g) were placed in an anesthesia machine and a small animal ventilator for continuous mechanical ventilation and anesthesia. Then, the skin surface of the left chest was disinfected and a thoracotomy through 3, 4 intercostal area was performed to expose the heart. left anterior descending artery (LAD) was occluded by tying a slipknot with 5-0 silk suture 1–2 mm from the lower edge of the left atrium. After 45 min, the slipknot was released to allow 3 day reperfusion. Sham-operated mice underwent the same surgical procedures as the IR group, including anesthesia, thoracotomy, and exposure of the heart, but the LAD was not ligated. All other experimental conditions, including ventilation, temperature control, and duration of surgery, were kept identical to the IR group.

### RNA-sequencing

Each group consisted of eight biologically independent samples, each biological replicate was processed, sequenced, and analyzed independently. While we did not perform separate library preparations or sequencing runs for the same RNA extract. Total RNA was extracted from heart tissue using the TRIzol method. After sample quality control, library construction was performed, with the main workflow as follows: First, mRNA enrichment is performed, followed by random fragmentation of the mRNA. Next, cDNA strands are synthesised and purified, modified, and ligated. Finally, PCR enrichment is performed to construct the cDNA library. After library quality control (LQC) approval, different libraries are pooled according to the target sequencing data volume and loaded onto sequencing chips for sequencing using the Illumina platform. The raw sequencing data are filtered to obtain clean data, which is then aligned with the specified reference genome to generate mapped data. Differential expression analysis, functional annotation of differentially expressed genes, and functional enrichment analysis are performed based on gene expression levels across different samples or sample groups. The raw data were quality-controlled using FastQC. High-quality reads were aligned to the reference genome using the STAR aligner. Differential expression analysis was performed with the DESeq2 software. KEGG and GO enrichment analyzes, *etc*., were performed using the Metware Cloud.

### Widely target metabolomics sequencing

The samples are chopped and mixed, and 20 mg (± 1 mg) is weighed from multiple points into centrifuge tubes with corresponding numbers. After centrifugation, 400 μL of 70% methanol water internal standard extraction solution is added, and the mixture is shaken at 2,500 r/min for 5 min and left to stand on ice for 15 min. Centrifuge at 12,000 rpm for 10 min at 4 °C, transfer 300 μL of the supernatant to another corresponding centrifuge tube, and incubate at −20 °C for 30 min. At 4 °C, centrifuge at 12,000 rpm for 3 min, transfer 200 μL of the supernatant to the corresponding injection vial liner, and use for instrument analysis. The T3 column is a Waters ACQUITY UPLC HSS T3 C18 1.8 µm, 2.1 mm × 100 mm, and the HILIC column is a Waters ACQUITY UPLC BEH HILIC Column, 1.7 µm, 1 mm × 100 mm. The data acquisition instrumentation system primarily includes ultra-performance liquid chromatography (UPLC) and tandem mass spectrometry (MS/MS). All sample extracts were mixed in equal volumes to form QC samples. Metabolite quantification was performed using multiple reaction monitoring (MRM) mode on a triple quadrupole mass spectrometer. Mass spectrometry data were processed using Analyst 1.6.3 software.

### Molecular docking

The receptor proteins studied included Pla2g2d (UniProt ID: Q9UNK4), Lcn2 (UniProt ID: P80188), and GPR55 (UniProt ID: Q9Y2T6). All crystal structures were retrieved from the RCSB PDB database and filtered according to the following criteria to ensure quality: resolution ≤2.5 Å, R-free <0.3, sequence completeness >95%, and average B-factor <80 Å^2^. Protein preparation was performed using PyMOL 2.6 to remove water molecules and non-essential ligands (*e.g.*, phosphate ions) from the crystal structures. Polar hydrogen atoms were then added and Kollman charges assigned using AutoDockTools 1.5.6. The ligand itaconic acid was energy-minimized with the MMFF94 force field in Chem3D 20.0, followed by addition of all hydrogen atoms and assignment of Gasteiger charges in AutoDockTools 1.5.6. Molecular docking was carried out on the AutoDock Vina 1.1.2 platform. The docking grid was centered on the original co-crystallized ligand or known active site, with a grid size of 40 Å × 40 Å × 40 Å; all other parameters were kept at default. Finally, Discovery Studio 2019 and PyMOL 2.6 were used to visualize and analyze ligand-protein interactions and generate three-dimensional interaction diagrams.

### Molecular dynamics simulation

Molecular dynamics simulations of itaconic acid with Pla2g2d, Lcn2, and GPR55 were performed in GROMACS 2022. Force-field parameters were assigned using the built-in pdb2gmx tool and the AutoFF server. Systems were neutralized with gmx genion, and long-range electrostatics were treated with PME (1.0 nm cutoff). Bonds were constrained with SHAKE, and integration used a 1 fs time step (Verlet leapfrog). Each system was energy-minimized using 3,000 steps of steepest descent followed by 2,000 steps of conjugate gradient. To evaluate binding stability and conformational dynamics, the following metrics were calculated from the simulation trajectories: the root-mean-square deviation (RMSD) of the protein backbone (reflecting overall structural stability), the per-residue root-mean-square fluctuation (RMSF) (indicating local flexibility), hydrogen-bond occupancy (for persistent interactions), the radius of gyration (Rg) (describing global compactness), and the solvent-accessible surface area (SASA). All analyzes were performed using standard GROMACS tools to ensure reproducibility.

### Echocardiography

Mice were anaesthetized with isoflurane and underwent cardiac ultrasound examination using the Vevo3100 small animal ultrasound system. The mouse limbs were fixed on a temperature-controlled plate, an ultrasound coupling agent was applied, and short-axis B-mode ultrasound and M-mode ultrasound images were acquired at the level of the papillary muscles. Ultrasound data were analyzed using Vevo LAB 3.2.6.

### HE staining

Mouse hearts were fixed in 4% paraformaldehyde solution for 1–2 days, followed by dehydration, clearing, paraffin embedding, and sectioning into five µm slices. The sections were spread out, dried, and baked at 58 °C before being left at room temperature overnight to dry completely. Sections were stained using the HE staining protocol, mounted with neutral balsam, dried in a fume hood, and observed and photographed under an inverted microscope.

### Immunohistochemistry

Paraffin sections were baked at 60 °C, dewaxed with xylene for 10 min × 2 times, hydrated with graded ethanol to distilled water, and washed with PBST. The antigen retrieval solution was boiled in a microwave oven, then cooled and washed with PBST. Incubate with 3% H_2_O_2_ at room temperature for 10 min, wash, and block with goat serum for 60 min. Sections were incubated with diluted primary antibodies against inducible nitric oxide synthase (iNOS) (Affinity, Cat. No. AF0199) and ARG1 (Affinity, Cat. No. DF6657) at 4 °C overnight, then washed with PBST after warming. Add secondary antibody and incubate at room temperature for 30–60 min, wash, and develop with DAB. Stop under the microscope. Counterstain with hematoxylin, dehydrate and clear, then mount with neutral binder.

### Immunofluorescence

Paraffin sections were dewaxed and rehydrated, followed by blocking with goat serum for 1 h. Staining was performed using a multiplex immunofluorescence staining kit (AiFang, Cat. No. AFIHC034). The sections were first incubated overnight with the primary antibody against F4/80 (Proteintech, Cat. No. 28463-1-AP), washed three times with PBS (5 min each), and then incubated with Polymer-HRP goat anti-mouse/rabbit secondary antibody at room temperature for 30 min. After another three PBS washes (5 min each), tyramide signal amplification (TSA) fluorescent dye reaction solution was applied for 5 min, followed by three additional PBS washes. A multiplex immunofluorescence-specific antibody elution solution was applied to cover the samples for 20 min. The above steps were repeated for sequential fluorescence labeling of CCR2 (Cat. No. 16153-1-AP; Proteintech, Rosemont, IL, USA) and CD206 (Cat. No. 83485-1-RR; Proteintech, Rosemont, IL, USA). Finally, an antifade mounting medium containing DAPI was applied, coverslips were gently placed, and the sections were observed under a confocal microscope (SP8; Leica, Wetzlar, Germany).

### ELISA

Abdominal venous blood was collected from mice and placed into EDTA tubes, centrifuged at 4 °C, 3,500 RPM for 15 min, and the supernatant was stored at −80 °C. Serum biochemical indexes including IL10 (Jonlinbio, Cat. No. JLW20242), IL4 (CloudClone, Cat. No. SEA077Mu), cTnT (Jonlinbio, Cat. No. JL40538), CKMB (Mlbio, Cat. No. ml107303).

### qRT-PCR

Total RNA was extracted from the myocardial samples and cells using RNA Easy Fast Tissue/Cell Kit (Cat. No. DP451; Tiangen, Beijing, China). One microgram of total RNA from each sample was reverse-transcribed into cDNA using the PrimeScript™ RT reagent Kit with gDNA Eraser (Cat. No. RR047A; Takara, Shiga, Japan), according to the manufacturer’s instructions. The qRT-PCR was performed using a SYBR Green qPCR Reagent Kit (Cat. No. RR820A; Takara, Shiga, Japan). The used primers are listed in Table 1. The mRNA levels were normalized to *β*actin mRNA levels and analyzed by using the 2$\hat {}$(−ΔΔCt) method. A limitation of this analysis is that *β*actin expression may be altered under ischemia-reperfusion conditions, potentially affecting normalization.

**Table 1:** Forward and reverse primer sequences.

Primers	Forward	Reverse
Il1b	AATGACCTGTTCTTTGAAGTTGA	TGATGTGCTGCTGCGAGATTTGAAG
Tnfa	GAAAAGCAAGCAGCCAACCA	CGGATCATGCTTTCTGTGCTC
Il4	AGATGGATGTGCCAAACGTCCTCA	AATATGCGAAGCACCTTGGAAGCC
Il10	CCAAGCCTTATCGGAAATGA	TTTTCACAGGGGAGAAATCG
*β*actin	TACTGCTCTGGCTCCTAGCA	CGGACTCATCGTACTCCTGC

### Statistical analysis

All results were analyzed using GraphPad Prism 9.0 software (GraphPad Software, San Diego, CA, USA) and presented as means ± standard deviation. Group comparisons were conducted using one-way analysis of variance (ANOVA). *P*-values less than 0.05 were considered statistically significant. The following values were considered to be statistically significant: *P < 0.05, **P < 0.01, ***P < 0.001, ****P < 0.0001.

Differentially expressed genes (DEGs) and metabolites were identified using the following thresholds: Genes: —log2FoldChange— ≥ 1 and FDR < 0.05, Metabolites: Variable Importance in Projection (VIP) > 1 and —log2FoldChange— ≥ 0.585 (corresponding to 1.5-fold change) and *p*-value < 0.05 (Student’s *t* test). These thresholds were selected to balance sensitivity and specificity, in line with previous 4-OI omics studies (Zhu et al., 2026). For integrated transcriptome-metabolome correlation analysis, Pearson correlation coefficients were calculated. Correlations with —*r*— ≥ 0.8 and *p*-value < 0.05 were considered significant. This cutoff was chosen to identify strongly associated gene-metabolite pairs while reducing spurious associations. No multiple-testing correction was applied to the correlation *p*-values; therefore, these results were interpreted as exploratory associations.

## Results

### Transcriptomics reveals increased inflammation infiltration in myocardial IR

The statistical power of this experimental design, calculated in RNASeqPower is 0.88, the analysis was based on the following parameters: the variable to estimate was set to power, sequencing depth was 30, sample size was 8, coefficient of variation was 0.4, effect size was 2, and alpha was 0.05. The raw transcriptome sequencing data associated with this study have been deposited in the NCBI Sequence Read Archive under accession number PRJNA1314415. Sequencing quality control revealed that each sample yielded 56,424,648–74,527,364 clean reads, with average quality scores of Q20 and Q30 at 98.95% and 96.70%, respectively, indicating high-quality clean reads. This project utilised the Ensembl database reference genome, annotation version number: Mus_musculus.GRCm39.109, with an average alignment rate of 98.76% across the 16 samples. Pearson’s correlation coefficient analysis revealed that the transcriptomic reproducibility within the sham and IR groups was excellent, with *r* ≥ 0.95 (Fig. S1).

**Figure 1:**
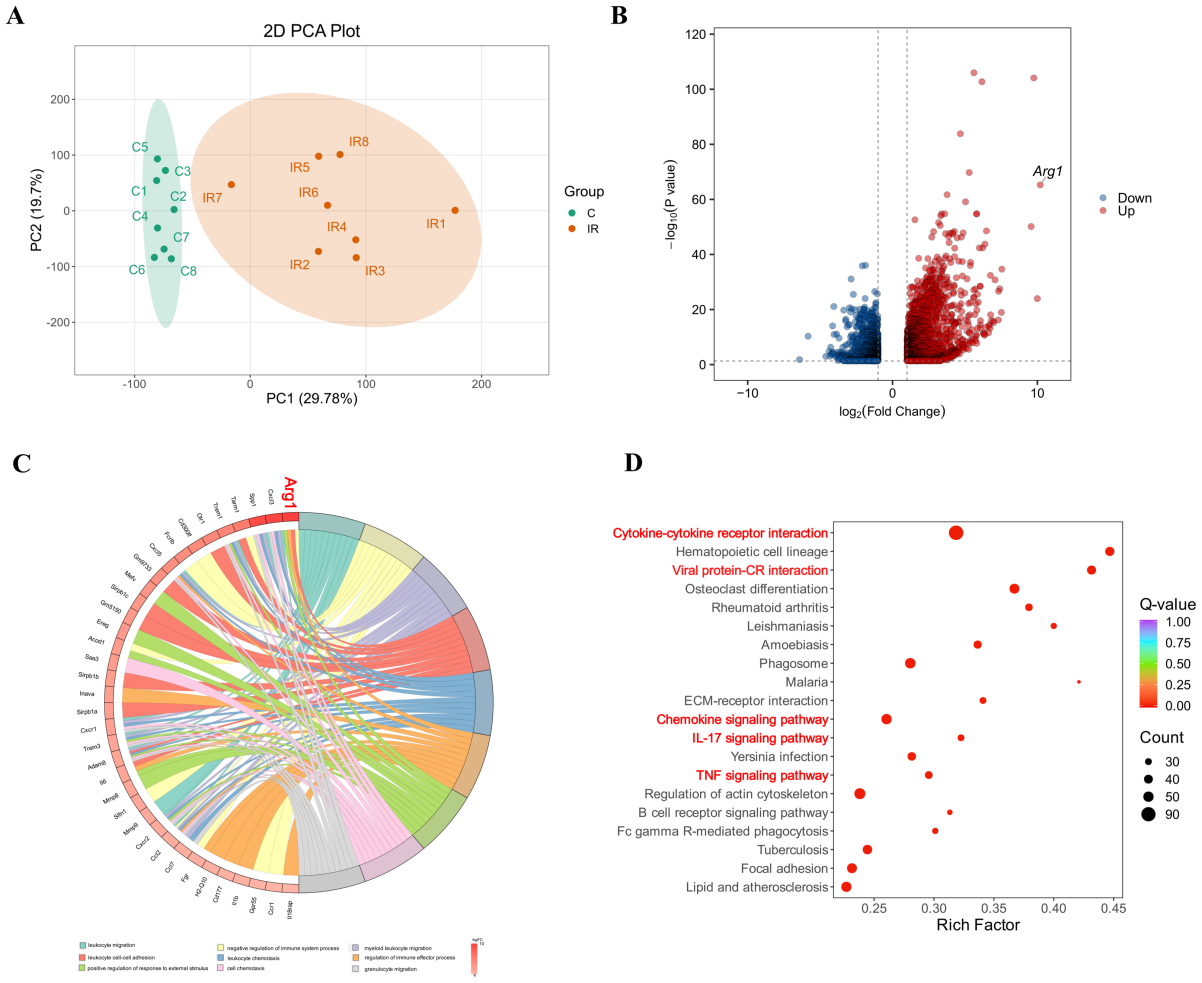
Transcriptomic analysis of myocardial ischemia-reperfusion injury in C57 mice. (A) Principal Component Analysis (PCA) plot of samples. (B) Volcano plot of differentially expressed genes. (C) KEGG enrichment chord plot. (D) GO enrichment dotplot.

Principal component analysis indicated significant differences between the sham and IR groups, with minimal differences within each group (Fig. 1A). Differential expression gene analysis was performed using DESeq2/edgeR, revealing 1,398 downregulated genes and 1,846 upregulated genes in the IR group. The volcano plot of differentially expressed genes (Fig. 1B) visually displays the overall distribution of differentially expressed genes in the sham and IR groups. The volcano plot reveals that the expression of the Arg1 gene is significantly upregulated. This high fold change should be interpreted cautiously, as it may reflect low baseline expression or normalization artifacts. Arg1 is a marker of M2 macrophages (Arlauckas et al., 2018) and is closely associated with M2 macrophage polarization (Li et al., 2022). This suggests that M2 macrophage infiltration increases 3 days after myocardial IR injury (Liu et al., 2025a), and increased M2 macrophage infiltration may be involved in the initiation of the anti-inflammatory mechanism after myocardial IR (Chen et al., 2025). The nine GO terms with the smallest *q*-values were selected to plot a GO enrichment chord plot. The Log_2_FC of Arg1 was 10.20. KEGG enrichment chord plot (Fig. 1C) revealed that Arg1 is closely associated with inflammation-related pathways, including leukocyte cell–cell adhesion, regulation of immune effector processes, and positive regulation of response to external stimuli (Hilgendorf, Frantz & Frangogiannis, 2024; Peet et al., 2020; Zhang et al., 2024). Among the KEGG enrichment analysis results, the 20 KEGG pathways with the lowest *q*-values were selected. It was found that pathways related to inflammation, such as cytokine-cytokine receptor interaction, viral protein-CR interaction, chemokine signaling pathway, IL-17 signaling pathway, and TNF signaling pathway (Fig. 1D), showed significant changes after myocardial IR (Hilgendorf, Frantz & Frangogiannis, 2024), indicating that inflammation plays a central role in the myocardial IR process. The Gene Set enrichment Analysis (GSEA) KEGG ridgeplot reveals upregulation of core genes enriched in inflammation-related pathways including the IL-17 signaling pathway, cytokine-cytokine receptor interaction, viral protein-cytokine receptor interaction, TNF signaling pathway, chemokine signaling pathway, NF-*κ*B signaling pathway, and natural killer cell-mediated cytotoxicity.

### Metabolomics identifies itaconic acid as a key metabolite in myocardial IR injury

A combination of UPLC-MS/MS detection platform, self-built database, and multivariate statistical analysis was used to study the metabolic differences between the sham group and the IR group (*n* = 8). The metabolomics data generated in this study have been deposited in the MetaboLights repository under the accession number MTBLS12934 and are publicly accessible *via* https://www.ebi.ac.uk/metabolights/editor/MTBLS12934/descriptors. Analyst was used to process the mass spectrometry data. The overlaid total ion chromatograms (TIC) of quality control samples (Fig. S2A) showed high overlap in the TIC curves of the metabolites. Pearson correlation analysis of the quality control samples (Fig. S2B) yielded an *r* value close to 1. The major metabolite categories detected in cardiac tissue were: amino acids and their metabolites (19.70%), organic acids and their derivatives (13.97%), glycerophospholipids (8.46%), nucleotides and their metabolites (8.1%), benzene and substituted derivatives (7.54%), heterocyclic compounds (7.14%), *etc*. (Fig. S2C).

**Figure 2:**
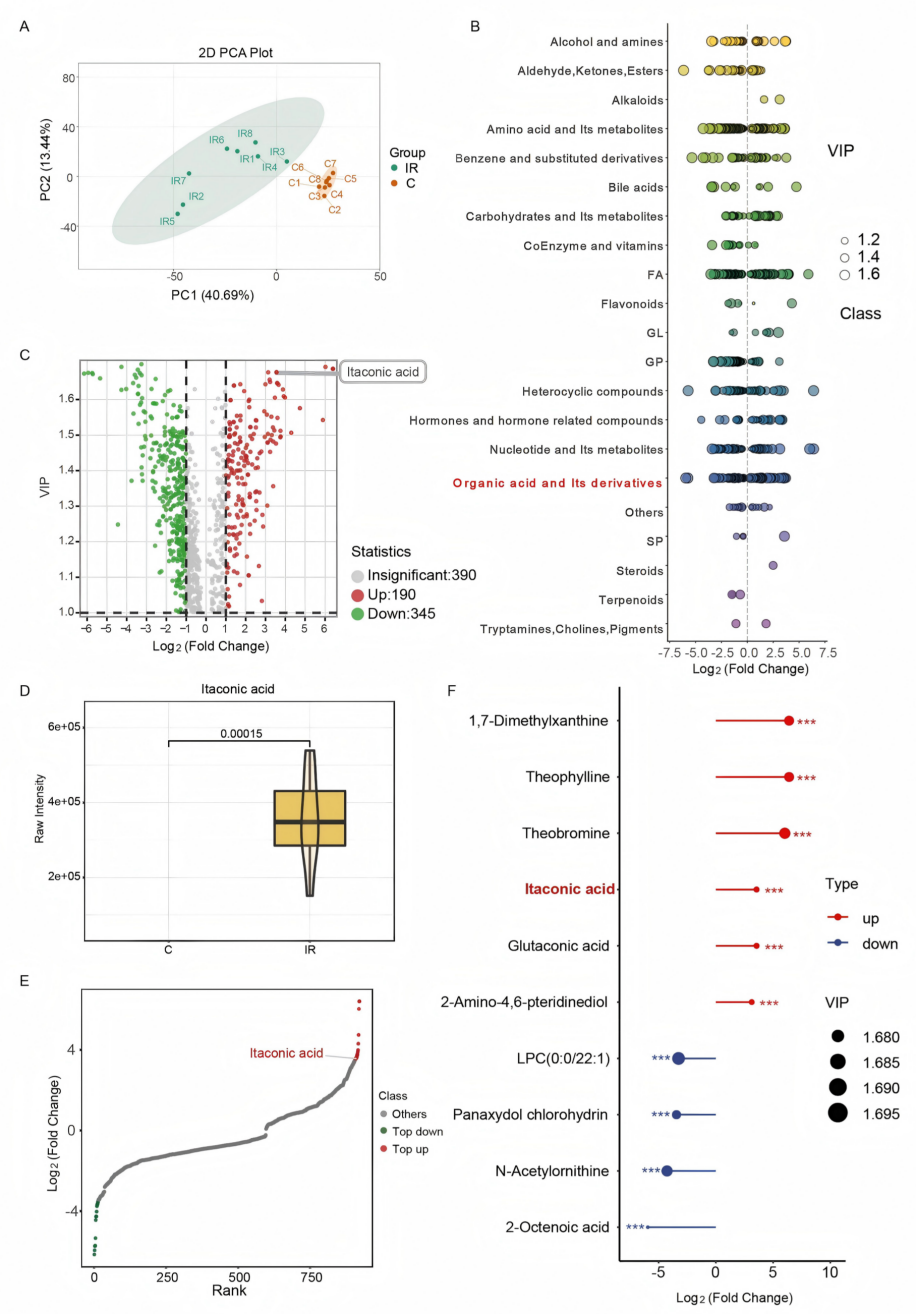
Metabolomics analysis of myocardial ischemia-reperfusion injury in C57 mice. (A) Principal Component Analysis (PCA) plot of samples. (B) Differential metabolite scatter plot. (C) Volcano plot of differentially expressed metabolites. (D) Relative content of itaconic acid violin chart. (E) Differential metabolite dynamic distribution plot. (F) Differential substances lollipop chart.

Principal component analysis (PCA) was performed on metabolites from the sham group and the IR group. As shown in Fig. 2A, metabolites from the two groups were separated on the first and second principal components, and there was significant variability in metabolites. Samples from the two groups clustered into separate categories, indicating high reproducibility and stability of the mass spectrometry data. The differential metabolite scatter plot (Fig. 2B) reveals significant differences among substances such as Alcohol and amines, amino acids and their metabolites, and organic acids and their derivatives. Among these, itaconic acid (Log_2_FC = 3.56) belongs to the organic acid and its derivatives category (Ye et al., 2024). Itaconic acid is a derivative of the TCA cycle intermediate cis-aconitate, produced by decarboxylation of cis-aconitate catalyzed by ACOD1, which is encoded by IRG1. Recent studies have shown that itaconic acid is an immunomodulatory metabolite that can alter immune cell function (Shan et al., 2025), but its role and mechanism in myocardial IR remain unclear. Volcano plots of different metabolites were generated based on VIP values and Log_2_FC values (Fig. 2C), showing 206 upregulated metabolites and 346 downregulated metabolites, among which itaconic acid (VIP = 1.68, Log_2_FC = 3.56) was significantly upregulated. The violin plot of the relative content (raw peak area) of itaconic acid (Fig. 2D) indicated that the content of itaconic acid was significantly increased in the IR group (*p* = 0.00015). The differential metabolite dynamic distribution plot (Fig. 2E) showed that itaconic acid (Log_2_FC = 3.56) was significantly upregulated in the IR group. The differential substances lollipop chart (Fig. 2F) reflects the VIP, FC, and *P*-value information of the difference substances. Through the difference substance lollipop plot, it can be observed that metabolites such as 1,7-Dimethylxanthine, theophylline, theobromine, itaconic acid, and glutaconic acid are significantly upregulated. In contrast, LPC (0-14:1), deoxycholic acid, and LPC (0:0/22:1) are downregulated.

### Integrated analysis of metabolomics and transcriptomics

**Figure 3:**
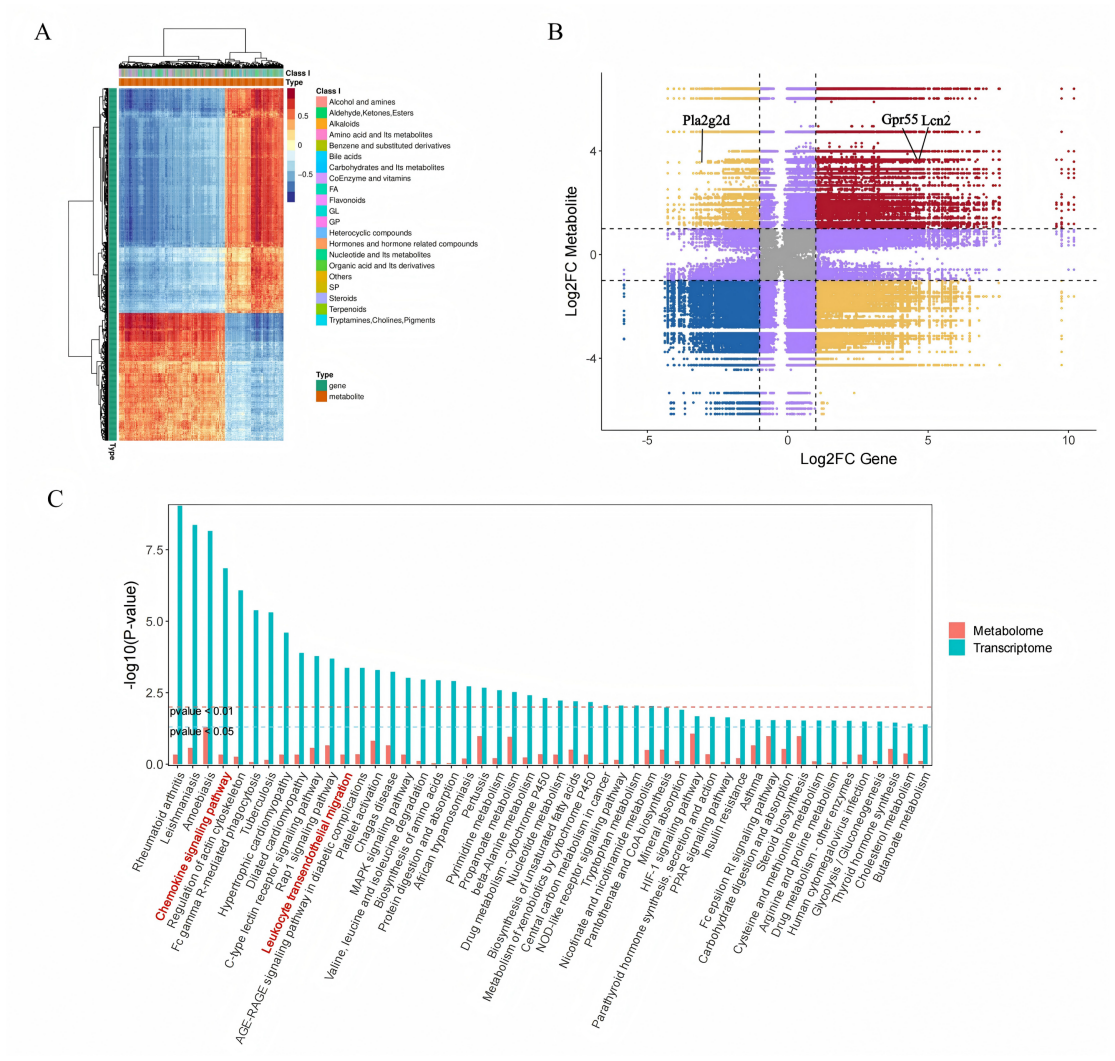
Integrated analysis of metabolomics and transcriptomics. (A) Clustered correlation heatmap of differentially expressed genes and differentially abundant metabolites. (B) Nine-quadrant plot for correlation analysis. (C) Bar plot of KEGG enrichment Analysis.

The Pearson correlation coefficient between genes and metabolites was calculated using the cor function in R. Correlation results with an absolute correlation coefficient greater than 0.8 and a *p*-value less than 0.05 were selected to plot a correlation cluster heat map (Fig. 3A). A nine-quadrant plot was used to display the fold change of genes and their corresponding metabolites with significant correlations. Among these, itaconic acid was correlated with the expression of genes associated with inflammatory infiltration, Pla2g2d, Lcn2, and Gpr55 (An et al., 2023; Miki et al., 2013). Recent studies have shown that Lcn2 is related to mechanisms of myocardial IR injury and inflammatory infiltration (Li et al., 2024), while Gpr55 is associated with inflammatory infiltration and post-myocardial infarction remodeling (Miao, Cheng & Guan, 2025; Puhl et al., 2021).

Based on the KEGG enrichment analysis results of differential metabolites and genes between the sham and myocardial IR groups, 50 KEGG pathways were identified that were commonly annotated in both groups (Fig. 3C). Through the KEGG enrichment analysis bar chart, pathways related to inflammation, such as the MAPK signaling pathway, chemokine signaling pathway, rheumatoid arthritis, and leukocyte transendothelial migration, were identified.

### Preliminary *in silico* analysis of possible interactions between itaconic acid and Pla2g2d, Lcn2, and Gpr55 proteins

**Figure 4:**
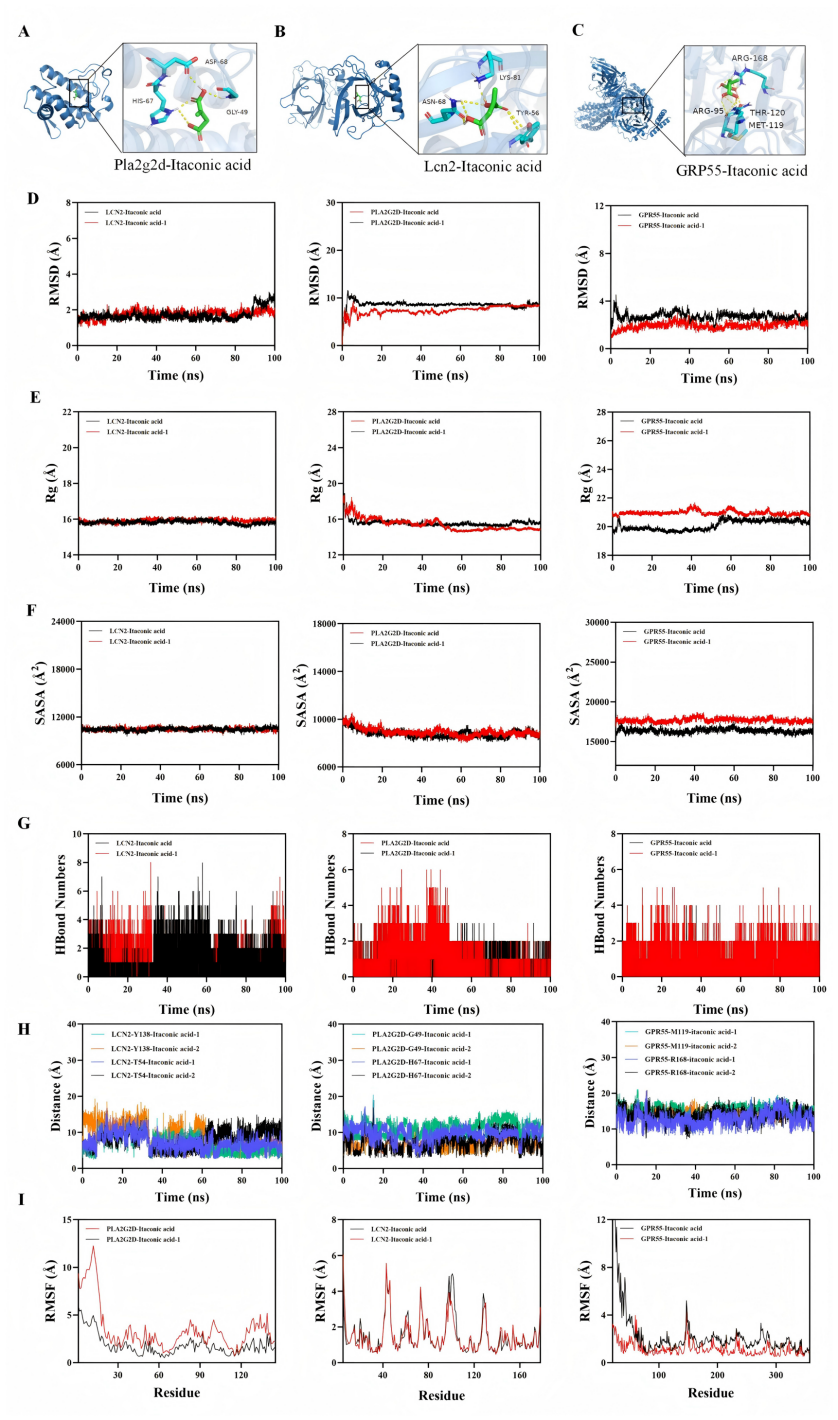
Molecular docking of itaconic acid with Pla2g2d, Lcn2, and Gpr55. (A), (B), and (C), respectively, show the molecular docking of Itaconic acid with Pla2g2d, Lcn2, and Gpr55. (D) Root-mean-square deviation (RMSD). (E) Radius of gyration (Rg). (F) Solvent-accessible surface area (SASA). (G) Hydrogen bonds (HBonds). (H) Key residue distance evolution. (I) Root mean square fluctuation (RMSF).

To further investigate the potential interactions identified in the integrated analysis, the inflammation-associated genes Pla2g2d, Lcn2, and Gpr55 (which were correlated with itaconic acid) were selected for molecular docking. Figures 4A, 4B, and 4C show the molecular docking results, with binding affinities of itaconic acid to Pla2g2d, Lcn2, and Gpr55 being −5.1, −5.5, and −5.3, respectively. This suggests that hydrogen bonds are formed between itaconic acid and the target proteins, although the binding affinities are relatively weak (approximately −5 kcal/mol).

To further investigate the dynamic changes of itaconic acid with Pla2g2d, Lcn2, and Gpr55 in complex physiological environments, molecular dynamics simulations were performed (the molecular dynamics simulation trajectories and related data have been deposited in the figshare repository under the DOI: 10.6084/m9.figshare.30226180). Figure 4D shows the RMSD plots of the complexes. The LCN2-itaconic acid complex exhibited stable fluctuations between 0–90 ns, followed by minor fluctuations after 90 ns, all within four Å. The PLA2G2D-itaconic acid complex showed relatively steady fluctuations during the simulation, eventually stabilizing around 10 Å. The GPR55-itaconic acid complex reached equilibrium after approximately 60 ns, with final fluctuations around 2.4 Å. Further analysis revealed slight fluctuations in the radius of gyration (Rg) and solvent accessible surface area (SASA) for the LCN2-itaconic acid, PLA2G2D-itaconic acid, and GPR55-itaconic acid complexes during the simulation, indicating minor expansion and contraction of these complexes (Figs. 4E–4F). Hydrogen bonds play a critical role in ligand-protein binding. The number of hydrogen bonds between the ligands and target proteins during the dynamics process is shown in Fig. 4G. For LCN2-itaconic acid, the number ranged from 0 to 8, with approximately four hydrogen bonds in most cases. For PLA2G2D-itaconic acid, the number ranged from 0 to 6, with around two hydrogen bonds in most cases. For GPR55-itaconic acid, the number ranged from 0 to 5, with approximately two hydrogen bonds in most cases. These hydrogen-bond patterns suggest possible transient or recurring ligand–protein contacts during the simulations. However, they do not demonstrate strong or functionally relevant binding, and their biological significance remains to be experimentally validated. As shown in Fig. 4H, all three complexes exhibited certain distance variations between the ligands and key amino acids. However, the distances gradually stabilized in the later stages of the simulation, indicating conformational changes during the dynamics process before reaching a stable state. It shows that the LCN2-itaconic acid, PLA2G2D-itaconic acid, and GPR55-itaconic acid complexes undergo certain conformational changes during the simulation but showed a tendency toward relative conformational stabilization during the later phase of the simulation. The root mean square fluctuation (RMSF) reflects the flexibility of amino acid residues in proteins. As shown in Fig. 4I, the RMSF values of the LCN2-itaconic acid complex were relatively low (mostly below four Å), those of the PLA2G2D-itaconic acid complex were relatively low (mostly below four Å), and those of the GPR55-itaconic acid complex were relatively low (mostly below three Å), suggesting relatively limited residue-level fluctuations under the simulated conditions (Dataset available at DOI: 10.6084/m9.figshare.30307108).

### 4-OI, a cell-permeable derivative of itaconic acid, inhibits inflammatory infiltration after myocardial IR

Integrated transcriptomic and metabolomic analyzes reveal that inflammation plays a critical role in early myocardial IR injury. Significant upregulation of Arg1, a biomarker of M2 macrophages, suggests substantial alterations in inflammatory cell infiltration post-injury. Recent evidence has identified itaconic acid as a key metabolic regulator in immune cells, particularly macrophages. Although native itaconic acid exhibits low membrane permeability, its two carboxyl groups enable esterification-derived derivatization to synthesize 4-OI. This cell-permeable analog shares biological functions with itaconic acid and undergoes intracellular conversion to the parent compound, thereby expanding the pharmaceutical potential of itaconic acid-based therapeutics (Liu et al., 2025b; Palsson-McDermott & O’Neill, 2025).

**Figure 5:**
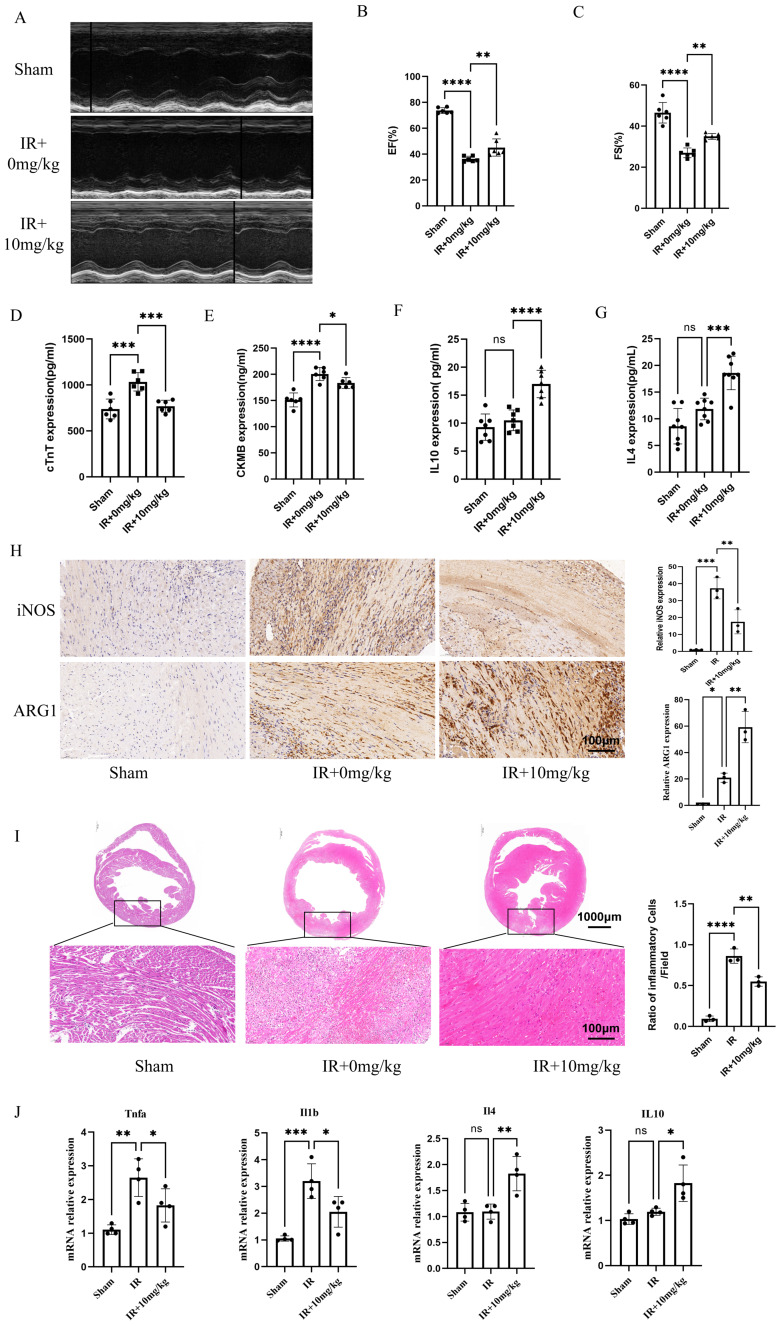
4-OI inhibits inflammatory infiltration after myocardial ischemia-reperfusion injury. (A) M-mode echocardiography of short axis in C57 mouse. (B) and (C) respectively show the ejection fraction (EF) and fractional shortening (FS) for each group. (D)–(G) respectively represent the levels of plasma cTnT, CKMB, IL10, and IL4; (H) Immunohistochemical Staining of iNOS and Arg1, scale bar = 100 µm. (I) Hematoxylin and eosin staining (H&E Staining), scale bar = 1,000 µm; scale bar = 100 µm. (J) Relative mRNA transcript levels of Tnfa, Il1b, Il4, and Il10 in mouse heart tissue. The asterisks indicate statistical significance as follows: *P < 0.05, **P < 0.01, ***P < 0.001, and ****P < 0.0001.

Following establishment of myocardial IR mouse models, 4-OI was administered *via* oral gavage at 10 mg/kg daily for three consecutive days. Figure 5A displays M-mode echocardiograms of IR mice treated with 4-OI. The 4-OI supplementation significantly increased ejection fraction (EF) and fractional shortening (FS) values (Figs. 5B–5C), while reducing plasma cTnT and CKMB levels (Figs. 5D–5E), suggesting improved cardiac function. To investigate 4-OI’s anti-inflammatory effects, we observed elevated plasma anti-inflammatory mediators IL10 and IL4 in 4-OI-treated IR mice (Figs. 5F–5G), suggesting a possible attenuation of systemic inflammation. Immunohistochemical staining showed reduced iNOS-associated staining and modestly increased ARG1-associated staining in myocardial tissue from 4-OI-treated mice compared with untreated IR mice. HE staining (Fig. 5I) showed well-aligned myocardial fibers and intact nuclei in sham controls, whereas IR hearts exhibited structural disruption with extensive neutrophil/monocyte infiltration in necrotic zones; 4-OI treatment markedly attenuated necrosis and inflammatory infiltration. Downregulated Tnfa/Il1b and upregulated Il4/Il10 mRNA expression in infarcted myocardium further supported an association with suppressed inflammation (Fig. 5J). These findings suggest that 4-OI may suppress post-infarction reperfusion inflammation.

**Figure 6:**
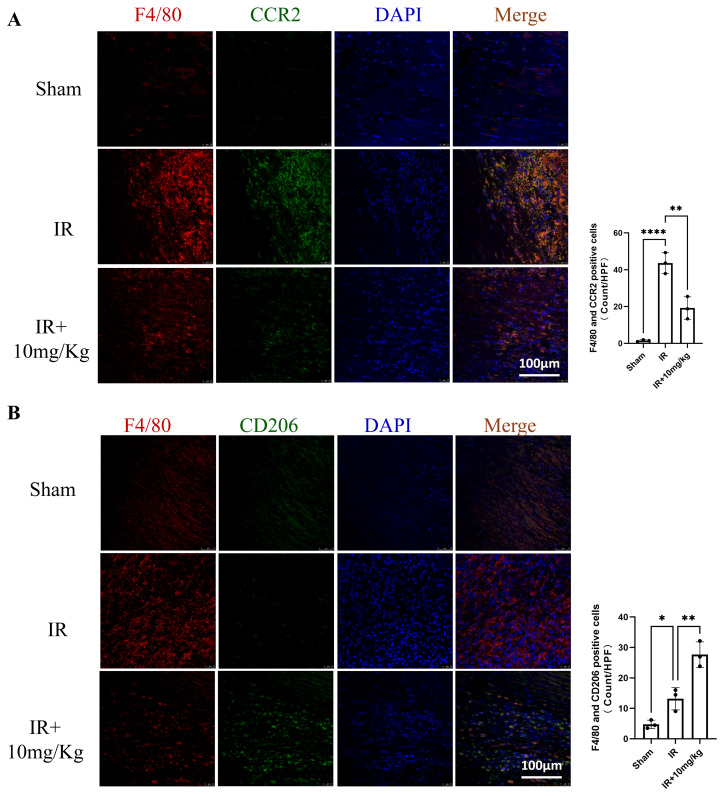
4-OI is associated with fewer F4/80+CCR2+ cells and more F4/80+CD206+ cells after myocardial IR. (A) Immunofluorescence analysis of the effect of 4-OI supplementation on F4/80^+^ CCR2^+^ cells. (B) Immunofluorescence analysis of the effect of 4-OI supplementation on F4/80^+^ CD206^+^ cells, scale bar = 100 µm. The asterisks indicate statistical significance as follows: *P < 0.05, **P < 0.01, and ****P < 0.0001.

To further characterize macrophage heterogeneity after myocardial IR injury, we performed immunofluorescence co-staining for F4/80 with CCR2 or CD206. The number of F4/80^+^CCR2^+^ cells was markedly increased in the IR group and significantly reduced by 4-OI treatment (Fig. 6A). In contrast, the number of F4/80^+^CD206^+^ cells was increased after IR injury and was further elevated following 4-OI treatment (Fig. 6B). Together, these findings suggest that 4-OI may be associated with changes in macrophage marker-defined populations in the injured myocardium, characterized by fewer F4/80^+^CCR2^+^ cells and more F4/80^+^CD206^+^ cells.

## Discussion

Transcriptomic analysis revealed that following myocardial IR injury, the expression of genes associated with inflammatory pathways, such as leukocyte cell–cell adhesion and the TNF signaling pathway, was upregulated in myocardial tissue, indicating that inflammation plays a central role in myocardial IR injury (Zuo et al., 2023). In this study, we observed a significant upregulation of Arg1 mRNA 3 days after myocardial IR injury. Extremely high Log_2_FC values were interpreted with caution, considering potential artifacts from low baseline expression or normalization, and require protein-level confirmation in future studies. Arg1 is commonly associated with an M2-like reparative phenotype; however, accumulating evidence highlights the substantial heterogeneity of cardiac macrophages following ischemic injury. Therefore, this upregulation should not be taken as definitive evidence of a canonical M2 macrophage population. Rather, it may represent a composite signal from multiple macrophage subsets engaged in both reparative and inflammatory programs. These findings underscore the importance of using multiple markers and single-cell approaches to deconvolute macrophage functional states in the injured heart (Li et al., 2023; Peet et al., 2020). Broad-target metabolomics screening revealed that itaconic acid levels were significantly upregulated after myocardial IR injury, which may be associated with increased levels of IL1b and TNFa after myocardial IR injury (He et al., 2024), as well as citrate accumulation in the TCA cycle (ElAzzouny et al., 2017). Transcriptome-metabolome joint analysis showed that itaconic acid showed exploratory correlations with inflammation-related genes such as Pla2g2d, Lcn2, and Gpr5. This correlation may provide mechanistic insights. However, the correlative nature of this approach does not permit causal inference, and these candidate interactions require independent experimental validation. Further molecular docking and molecular dynamics simulations provided preliminary *in silico* support for possible interactions between itaconic acid and Pla2g2d, Lcn2, and Gpr55. However, these computational observations do not confirm direct binding and require validation by experimental approaches such as surface plasmon resonance, isothermal titration calorimetry, pull-down assays, or cellular target-engagement assays. It should be noted that, although the false discovery rate (FDR) correction was applied to the transcriptomic differential expression analysis, no additional multiple-testing correction was performed for the metabolomic comparisons or the gene–metabolite correlation *p*-values. This implies that the metabolomic and integrated correlation results may contain false-positive associations, particularly given the exploratory nature of this multi-omics study and the modest sample size. Accordingly, these findings should be interpreted as hypothesis-generating rather than confirmatory. Independent validation in separate cohorts and through targeted experimental assays will be necessary to confirm key candidate associations.

*In vivo*, itaconic acid is an immunometabolite that is significantly upregulated in macrophages upon inflammatory stimulation, derived from the conversion of cis-aconitate in the tricarboxylic acid cycle by aconitate decarboxylase encoded by IRG1. 4-OI, a cell-permeable derivative of itaconic acid, is the most commonly used tool compound for studying the biological functions of itaconic acid. In a myocardial IR mouse model, supplementation with 4-OI significantly increased EF and FS values and decreased serum cTnT and CKMB levels, indicating that 4-OI can improve myocardial IR injury. After supplementation with 4-OI, circulating IL4 and IL10 levels increased. HE staining showed reduced inflammatory cell infiltration in the myocardial IR border zone. Concurrently, mRNA expression levels of Il1b and Tnfa decreased in myocardial tissue. In contrast, mRNA expression levels of Il4 and Il10 increased, suggesting that 4-OI improves myocardial IR injury by inhibiting inflammatory infiltration. In addition, because *β*-actin was used as the internal reference gene for qRT-PCR normalization, potential changes in *β*-actin expression under ischemia-reperfusion conditions may affect the interpretation of qRT-PCR results. Immunohistochemical staining showed reduced iNOS-associated staining and modestly increased Arg1-associated staining after 4-OI treatment. Because these markers alone are insufficient to define macrophage subpopulations, we further characterized macrophage heterogeneity by immunofluorescence. CCR2-positive macrophages, primarily derived from monocytes infiltrating tissues, promote the NLRP3/caspase-1/IL1b-mediated inflammatory cascade, thereby exacerbating myocardial IR injury (Heo et al., 2019); during the repair phase after myocardial IR, CD206-positive macrophages become the predominant macrophage subset in the heart, improving myocardial IR injury by secreting anti-inflammatory mediators such as TGF*β* and IL10 (Li et al., 2023). 4-OI supplementation was associated with reduced F4/80^+^CCR2^+^ cells and increased F4/80^+^CD206^+^ cells in cardiac tissue. These correlative findings raise the possibility that itaconic acid may influence macrophage recruitment or phenotypic balance. However, CCR2 and CD206 are not subset-specific markers; therefore, these findings should be interpreted as changes in marker-defined macrophage-associated populations rather than definitive evidence of recruited inflammatory macrophages or resident reparative macrophages. In the absence of flow cytometric validation, lineage tracing, single-cell RNA sequencing, or macrophage-specific functional experiments, the ontogeny, functional state, and causal contribution of these cells to 4-OI-mediated cardioprotection remain to be determined (Bajpai et al., 2019).

Notably, previous studies have demonstrated that 4-OI promotes ischemic angiogenesis through ERK1/2-mediated activation of VEGF*α* and VEGFR2 (Yang et al., 2025). In the present study, 4-OI treatment was associated with an increased proportion of F4/80^+^CD206^+^ macrophages in the injured myocardium. Given that CD206-positive macrophages are known to secrete pro-angiogenic factors such as VEGF, this shift may provide upstream cues that contribute to the ERK-dependent angiogenic response. Furthermore, these macrophages produce anti-inflammatory cytokines (*e.g.*, IL10) and are linked to reduced pro-inflammatory chemokine accumulation, potentially limiting excessive neutrophil activation and subsequent NET formation-a mechanism previously implicated in the cardioprotective effects of 4-OI in heart transplantation (Tian et al., 2025). Although these findings suggest a possible interplay between macrophage heterogeneity, angiogenic signaling, and immunomodulation, it must be emphasized that our data do not directly test these mechanistic connections. Thus, further experimental validation is required to confirm these hypothesized relationships.

In summary, this study further demonstrates that 4-OI ameliorates myocardial IR injury in mice, consistent with previous reports showing its protective effects in this setting (Ku, Shen & Cheng, 2022; Yang et al., 2025). Extending beyond these earlier findings, our study reveals that 4-OI was associated with reduced inflammatory infiltration after myocardial IR injury, together with changes in macrophage marker-defined populations in the injured myocardium. However, this study did not explore the specific mechanisms by which 4-OI regulates macrophages following myocardial IR injury. Whether this effect is mechanistically driven by macrophage phenotypic shifts, or occurs independently, remains to be directly tested.

## Conclusions

In this study, integrated transcriptomic and metabolomic analyzes identified itaconic acid as a myocardial IR injury-associated immunometabolite linked to inflammatory pathways and candidate inflammation-related genes. *In vivo*, 4-OI, a cell-permeable derivative of itaconic acid, was associated with improved cardiac function, reduced injury-related markers, and attenuated inflammatory infiltration after myocardial IR injury. 4-OI treatment was also associated with reduced F4/80^+^CCR2^+^ cells and increased F4/80^+^CD206^+^ cells in the injured myocardium, suggesting changes in selected macrophage marker-defined populations rather than definitive macrophage subset assignment. These findings suggest that changes in the inflammatory microenvironment and macrophage-associated marker-defined populations may be involved in the beneficial effects observed after 4-OI treatment. However, the present study does not establish macrophage modulation as the causal mechanism responsible for cardioprotection, and the relationship between these macrophage-associated changes and previously reported ERK-mediated angiogenic pathways requires direct experimental validation.

## Supplemental Information

10.7717/peerj.21525/supp-1 Figure S1 Gene Set Enrichment Analysis (GSEA) KEGG Ridgeplot

10.7717/peerj.21525/supp-2 Figure S2 Widely Targeted Metabolomics Data Assessment (A) Overlaid Total Ion Chromatograms (TIC) of Quality Control Samples. (B) Quality Control Samples correlation. (C) Donut Plot of Metabolite Class Distribution.

10.7717/peerj.21525/supp-3 Data S1 Supplementary data

10.7717/peerj.21525/supp-4 Supplemental Information 4 ARRIVE checklist

10.7717/peerj.21525/supp-5 Supplemental Information 5 MIQE Checklist

## Additional Information and Declarations

### Data Availability

The following information was supplied regarding data availability: The raw measurements are available in the Supplementary Files. The transcriptomics data is available at NCBI: PRJNA1314415. The metabolomics data is available at MetaboLights: MTBLS12934.

## References

[ref-1] An HS, Yoo JW, Jeong JH, Heo M, Hwang SH, Jang HM, Jeong EA, Lee J, Shin HJ, Kim KE, Shin MC, Roh GS (2023). Lipocalin-2 promotes acute lung inflammation and oxidative stress by enhancing macrophage iron accumulation. International Journal of Biological Sciences.

[ref-2] Arlauckas SP, Garren SB, Garris CS, Kohler RH, Oh J, Pittet MJ, Weissleder R (2018). Arg1 expression defines immunosuppressive subsets of tumor-associated macrophages. Theranostics.

[ref-3] Bajpai G, Bredemeyer A, Li W, Zaitsev K, Koenig AL, Lokshina I, Mohan J, Ivey B, Hsiao HM, Weinheimer C, Kovacs A, Epelman S, Artyomov M, Kreisel D, Lavine KJ (2019). Tissue resident CCR2− and CCR2+ cardiac macrophages differentially orchestrate monocyte recruitment and fate specification following myocardial injury. Circulation Research.

[ref-4] Chen DX, Feng YY, Wang HY, Lu CH, Liu DZ, Gong C, Xue Y, Na N, Huang F (2025). Metrnl ameliorates myocardial ischemia-reperfusion injury by activating AMPK-mediated M2 macrophage polarization. Molecular Medicine.

[ref-5] ElAzzouny M, Tom CT, Evans CR, Olson LL, Tanga MJ, Gallagher KA, Martin BR, Burant CF (2017). Dimethyl itaconate is not metabolized into itaconate intracellularly. Journal of Biological Chemistry.

[ref-6] He R, Zuo Y, Yi K, Liu B, Song C, Li N, Geng Q (2024). The role and therapeutic potential of itaconate in lung disease. Cellular & Molecular Biology Letters.

[ref-7] Heo GS, Kopecky B, Sultan D, Ou M, Feng G, Bajpai G, Zhang X, Luehmann H, Detering L, Su Y, Leuschner F, Combadière C, Kreisel D, Gropler RJ, Brody SL, Liu Y, Lavine KJ (2019). Molecular imaging visualizes recruitment of inflammatory monocytes and macrophages to the injured heart. Circulation Research.

[ref-8] Hilgendorf I, Frantz S, Frangogiannis NG (2024). Repair of the infarcted heart: cellular effectors, molecular mechanisms and therapeutic opportunities. Circulation Research.

[ref-9] Ku HC, Shen TC, Cheng CF (2022). The potential of using itaconate as treatment for inflammation-related heart diseases. Tzu Chi Medical Journal.

[ref-10] Li L, Cao J, Li S, Cui T, Ni J, Zhang H, Zhu Y, Mao J, Gao X, Midgley AC, Zhu M, Fan G (2023). M2 macrophage-derived sEV regulate pro-inflammatory CCR2(+) macrophage subpopulations to favor post-AMI cardiac repair. Advanced Science.

[ref-11] Li P, Chen J, Wang M, Wang Q, Liu X (2024). High-fat diet-induced LCN2 exacerbates myocardial ischemia-reperfusion injury by enhancing platelet activation. Molecular Medicine Reports.

[ref-12] Li H, Zhu J, Xu YW, Mou FF, Shan XL, Wang QL, Liu BN, Ning K, Liu JJ, Wang YC, Mi JX, Wei X, Shao SJ, Cui GH, Lu R, Guo HD (2022). Notoginsenoside R1-loaded mesoporous silica nanoparticles targeting the site of injury through inflammatory cells improves heart repair after myocardial infarction. Redox Biology.

[ref-13] Liao X, Tang M, Li J, Guo R, Zhong C, Chen X, Zhang X, Mo H, Que D, Yu W, Song X, Li H, Cai Y, Yang P (2025). Acid-triggered cascaded responsive supramolecular peptide alleviates myocardial ischemia–reperfusion injury by restoring redox homeostasis and protecting mitochondrial function. Advanced Healthcare Materials.

[ref-14] Liu G, Dai Y, Fu C, Lv X, Qin J, Xie J (2025a). Macrophage polarization in myocardial ischemia–reperfusion injury: pathophysiology and therapeutic targets. Drug Design, Development and Therapy.

[ref-15] Liu N, Jiang Y, Xiu Y, Tortelote GG, Xia W, Wang Y, Li Y, Shi S, Han J, Vidoudez C, Niamnud A, Kilgore MD, Zhou D, Shi M, Graziose SA, Fan J, Katakam PVG, Dumont AS, Wang X (2025b). Itaconate restrains acute proinflammatory activation of microglia after traumatic brain injury in mice. Science Translational Medicine.

[ref-16] McGettrick AF, Bourner LA, Dorsey FC, O’Neill LAJ (2024). Metabolic messengers: itaconate. Nature Metabolism.

[ref-17] Miao Y, Cheng YY, Guan W (2025). GPR55: Physiological functions and therapeutic potential in depression. Biochemical Pharmacology.

[ref-18] Miki Y, Yamamoto K, Taketomi Y, Sato H, Shimo K, Kobayashi T, Ishikawa Y, Ishii T, Nakanishi H, Ikeda K, Taguchi R, Kabashima K, Arita M, Arai H, Lambeau G, Bollinger JM, Hara S, Gelb MH, Murakami M (2013). Lymphoid tissue phospholipase A2 group IID resolves contact hypersensitivity by driving antiinflammatory lipid mediators. Journal of Experimental Medicine.

[ref-19] Mills EL, Ryan DG, Prag HA, Dikovskaya D, Menon D, Zaslona Z, Jedrychowski MP, Costa ASH, Higgins M, Hams E, Szpyt J, Runtsch MC, King MS, McGouran JF, Fischer R, Kessler BM, McGettrick AF, Hughes MM, Carroll RG, Booty LM, Knatko EV, Meakin PJ, Ashford MLJ, Modis LK, Brunori G, Sévin DC, Fallon PG, Caldwell ST, Kunji ERS, Chouchani ET, Frezza C, Dinkova-Kostova AT, Hartley RC, Murphy MP, O’Neill LA (2018). Itaconate is an anti-inflammatory metabolite that activates Nrf2 *via* alkylation of KEAP1. Nature.

[ref-20] Palsson-McDermott EM, O’Neill LAJ (2025). Gang of 3: how the Krebs cycle-linked metabolites itaconate, succinate, and fumarate regulate macrophages and inflammation. Cell Metabolism.

[ref-21] Peet C, Ivetic A, Bromage DI, Shah AM (2020). Cardiac monocytes and macrophages after myocardial infarction. Cardiovascular Research.

[ref-22] Puhl SL, Hilby M, Kohlhaas M, Keidel LM, Jansen Y, Hristov M, Schindler J, Maack C, Steffens S (2021). Haematopoietic and cardiac GPR55 synchronize post-myocardial infarction remodelling. Scientific Reports.

[ref-23] Shan M, Zhang S, Luo Z, Deng S, Ran L, Zhou Q, Wan H, Ye J, Qian C, Fan X, Feng Y, Morse DW, Herrmann J, Li Q, Guo Z, Wang F (2025). Itaconate promotes inflammatory responses in tissue-resident alveolar macrophages and exacerbates acute lung injury. Cell Metabolism.

[ref-24] Tian H, Xiong Y, Zhan J, Lu Z, Zhang Y, Leng Y, Huang Q, Xia Z (2025). Inhibition of macrophage ARID3A alleviates myocardial ischemia-reperfusion injury after heart transplantation by reducing THBS1/CD47 signaling-mediated neutrophil extracellular traps formation. Advanced Science.

[ref-25] Tsao CW, Aday AW, Almarzooq ZI, Alonso A, Beaton AZ, Bittencourt MS, Boehme AK, Buxton AE, Carson AP, Commodore-Mensah Y, Elkind MSV, Evenson KR, Eze-Nliam C, Ferguson JF, Generoso G, Ho JE, Kalani R, Khan SS, Kissela BM, Knutson KL, Levine DA, Lewis TT, Liu J, Loop MS, Ma J, Mussolino ME, Navaneethan SD, Perak AM, Poudel R, Rezk-Hanna M, Roth GA, Schroeder EB, Shah SH, Thacker EL, Van Wagner LB, Virani SS, Voecks JH, Wang NY, Yaffe K, Martin SS (2022). Heart Disease and Stroke Statistics-2022 update: a report from the American Heart Association. Circulation.

[ref-26] Wang X, Yan X, Mang G, Chen Y, Liu S, Sui J, Tong Z, Wang P, Cui J, Yang Q, Zhang Y, Wang D, Sun P, Song W, Jin Z, Shi M, Zhao P, Yang J, Liu M, Wang N, Chen T, Ji Y, Yu B, Zhang M (2025). S100a9 lactylation triggers neutrophil trafficking and cardiac inflammation in myocardial ischemia/reperfusion injury. Journal of Clinical Investigation.

[ref-27] Xiang Q, Yi X, Zhu XH, Wei X, Jiang DS (2024). Regulated cell death in myocardial ischemia-reperfusion injury. Trends in Endocrinology & Metabolism.

[ref-28] Yang J, Duan C, Wang P, Zhang S, Gao Y, Lu S, Ji Y (2025). 4-octyl itaconate alleviates myocardial ischemia-reperfusion injury through promoting angiogenesis *via* ERK signaling activation. Advanced Science.

[ref-29] Ye D, Wang P, Chen LL, Guan KL, Xiong Y (2024). Itaconate in host inflammation and defense. Trends in Endocrinology & Metabolism.

[ref-30] Zhang M, Liu Q, Meng H, Duan H, Liu X, Wu J, Gao F, Wang S, Tan R, Yuan J (2024). Ischemia-reperfusion injury: molecular mechanisms and therapeutic targets. Signal Transduction and Targeted Therapy.

[ref-31] Zhu G, Ye L, Song Y, Chen Y, Cai H, Abuduxukuer Z, Zhu L, Zeng Y, Zhu W, Ye D, Song Y, Wang P, Jin M, Wang J (2026). Itaconate modulates neutrophil homeostasis to ameliorate airway inflammation in diesel exhaust particles-exacerbated asthma *via* inhibiting NETs formation. International Journal of Biological Sciences.

[ref-32] Zuo W, Sun R, Ji Z, Ma G (2023). Macrophage-driven cardiac inflammation and healing: insights from homeostasis and myocardial infarction. Cellular & Molecular Biology Letters.

