# Supplementary figures and images for: Integrated transcriptomic and metabolomic analysis reveals the regulatory role of itaconic acid in inflammatory infiltration during myocardial ischemia-reperfusion injury
Source: PeerJ. 2026 Jul 17;14:e21525. doi: 10.7717/peerj.21525 (PMC13383959; doi:10.7717/peerj.21525)

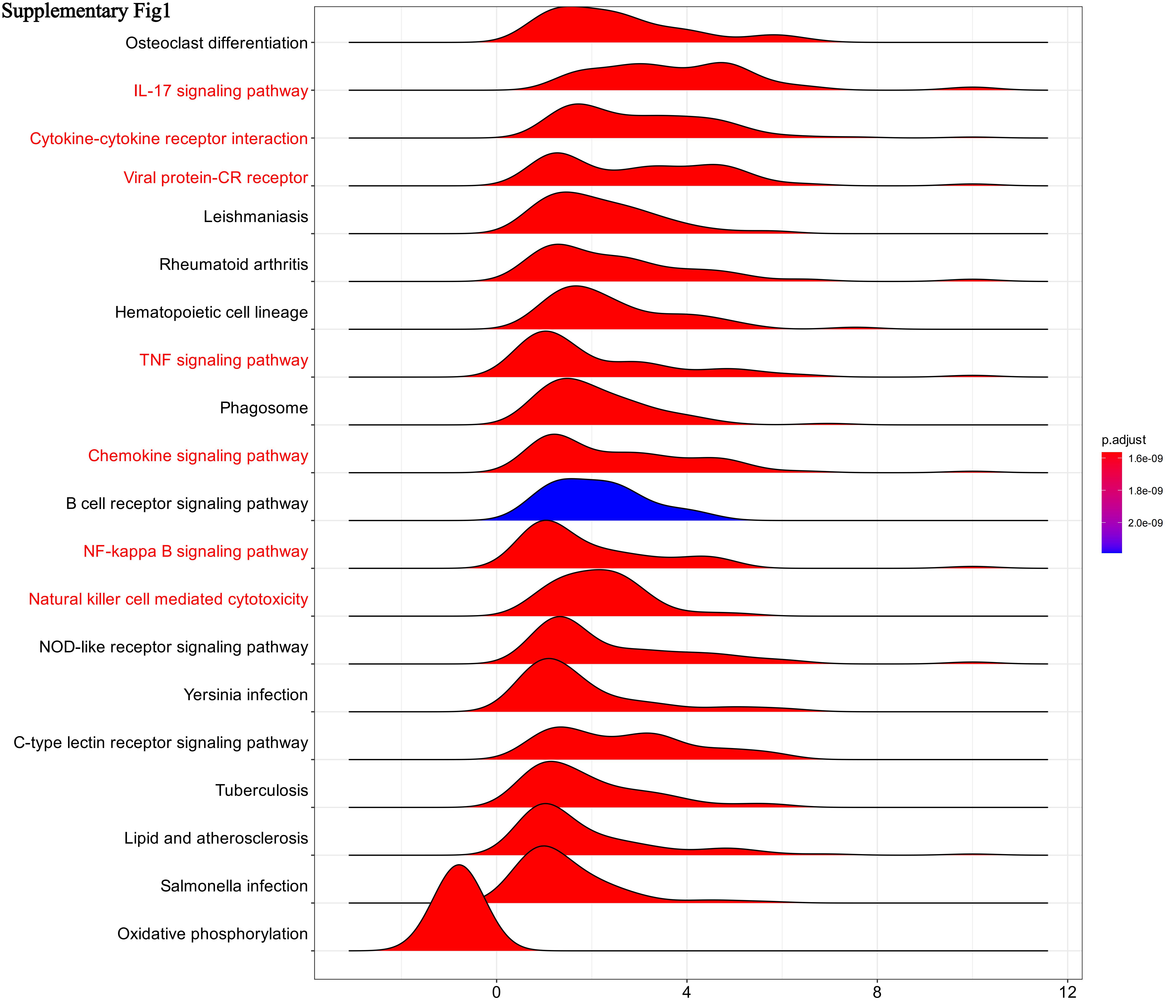

Supplement: Figure S1 [file peerj-14-21525-s001.jpg]

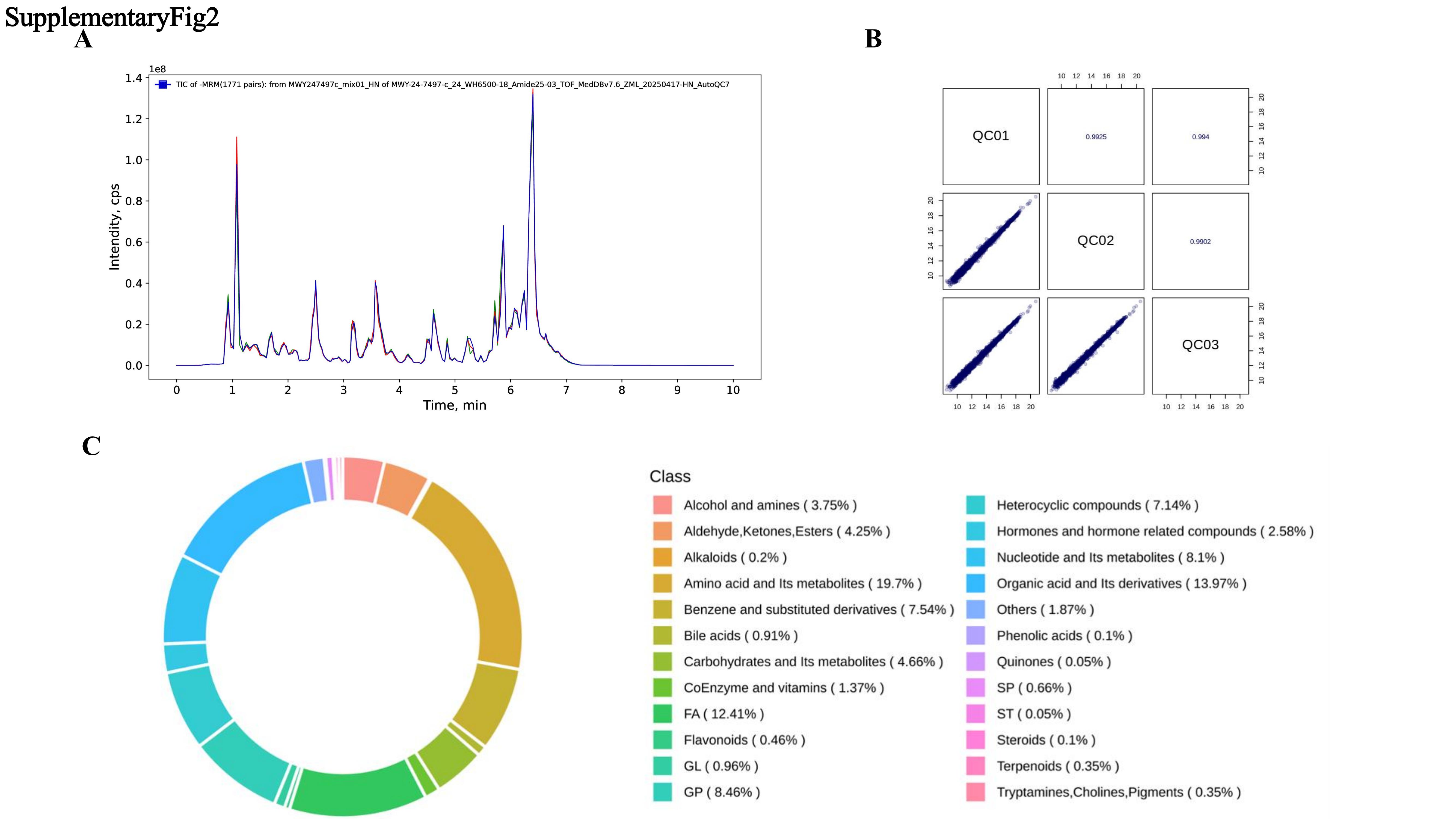

Supplement: Figure S2 — (A) Overlaid Total Ion Chromatograms (TIC) of Quality Control Samples. (B) Quality Control Samples correlation. (C) Donut Plot of Metabolite Class Distribution. [file peerj-14-21525-s002.jpg]
